# Use of TYK2 inhibitor to relieve reactive granulomatous dermatitis due to myelodysplastic syndrome

**DOI:** 10.1016/j.jdcr.2023.08.009

**Published:** 2023-08-19

**Authors:** Christina Huang, Adar Berghoff, Jack L. Arbiser

**Affiliations:** a Sidney Kimmel Medical College, Thomas Jefferson University, Philadelphia, Pennsylvania; b Metroderm/United Dermatology Partners, Atlanta, Georgia

**Keywords:** Granuloma formation, myelodysplastic syndrome, TYK2 Inhibition

## Introduction

Reactive granulomatous dermatitis (RGD) may occur in response to a variety of underlying etiologies, including autoimmune conditions, medications, and malignancy. Rarely, RGD may be a presenting sign of myelodysplastic syndrome or myeloid leukemia, and recognition of this cutaneous manifestation is essential so that patients can be evaluated and treated accordingly.^1^ The treatment of RGD centers around managing the underlying condition, although other treatments, including topical/oral corticosteroids, hydroxychloroquine, tetracycline antibiotics, methotrexate, cyclosporine, dapsone, and tumor necrosis factor-α inhibitors have been utilized with varying efficacy.^2^ In our case report, a patient presenting with RGD due to myelodysplastic syndrome experienced rapid relief from the cutaneous manifestation through treatment with deucravactinib, a systemic protein tyrosine kinase 2 (TYK2) inhibitor. Effective resolution of RGD in our patient demonstrates the potential utility of systemic TYK2 inhibition in the treatment of this condition. Furthermore, TYK2 inhibitors may play a role in treating other granulomatous disorders, including sarcoidosis or diffuse granuloma annulare.^3^

## Case report

**Fig 1:**
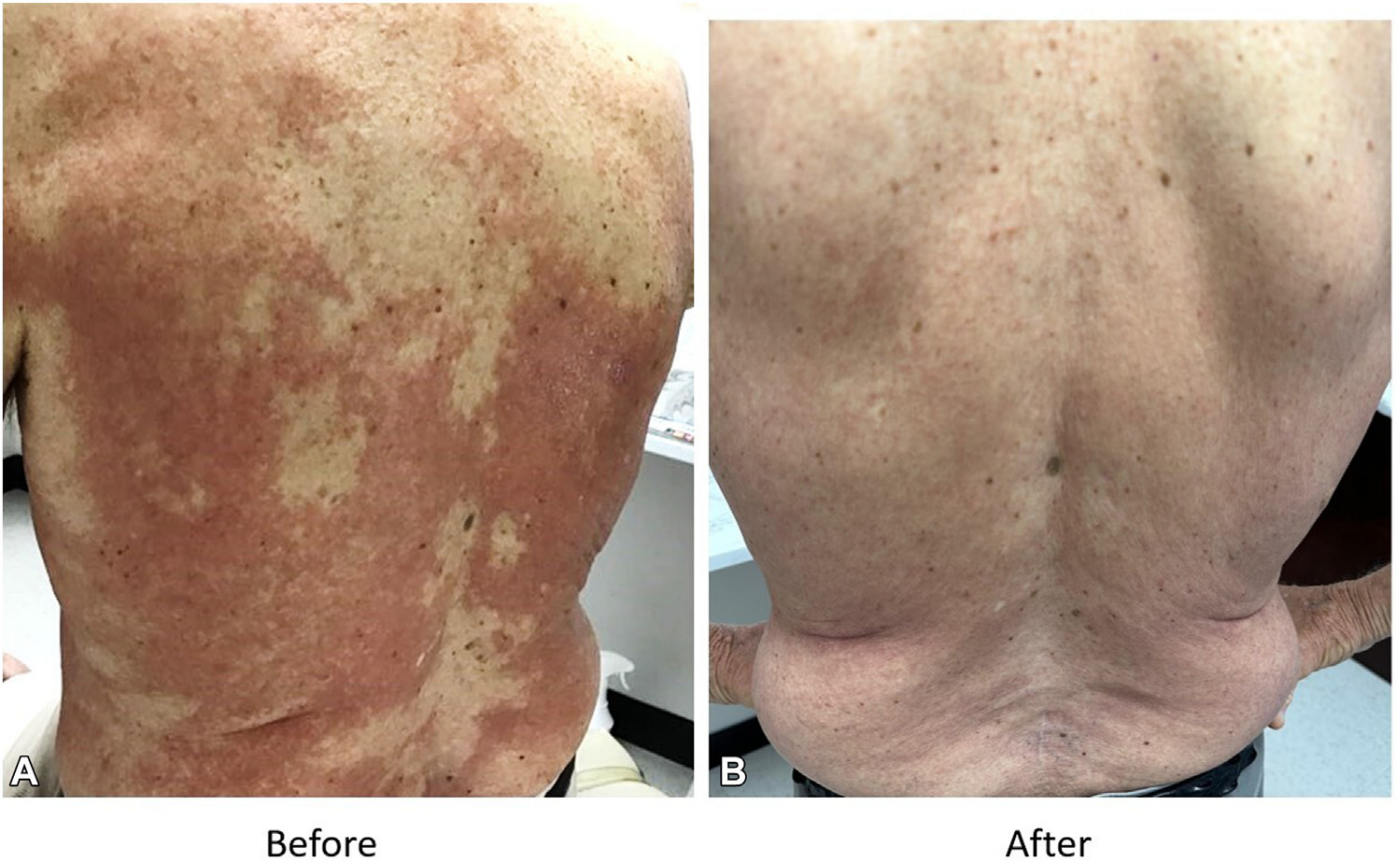
Reactive granulomatous dermatitis. **A,** Patient presenting before treatment with a 5-month history of diffuse, pruritic rash with multiple smooth flat plaques, which were deeply erythematous, compared with (**B**) patient presenting at follow up appointment a month later after 21 days of treatment with deucravacitinib

A 78-year-old White male patient presented with a 5-month history of a diffuse, pruritic eruptions on the back, chest, and legs that were unresponsive to topical corticosteroids (triamcinolone 0.1% and clobetasol 0.05%). Physical examination revealed multiple smooth flat plaques that were deeply erythematous (Fig 1, *A*).

**Fig 2:**
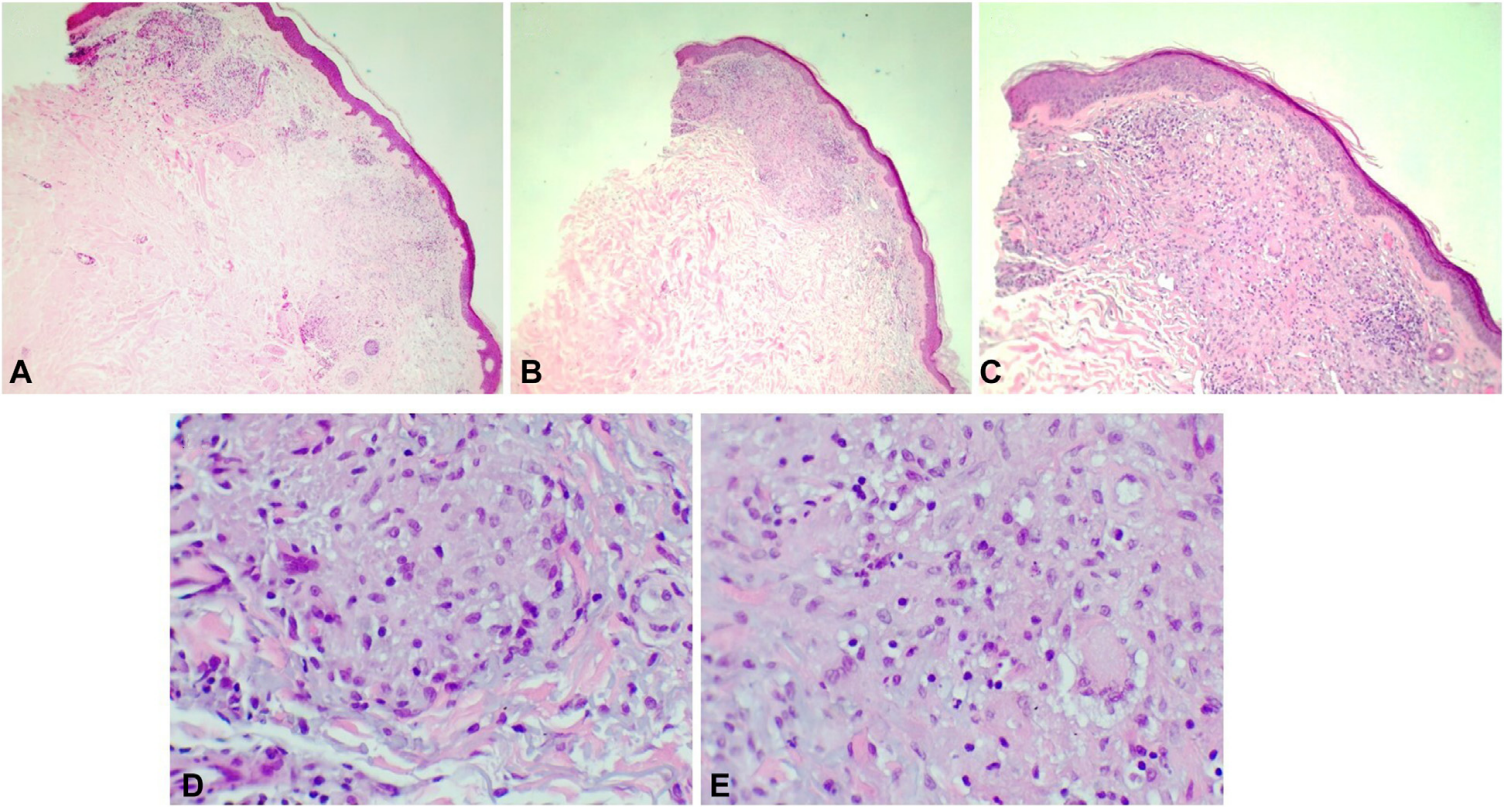
Reactive granulomatous dermatitis. Hematoxylin-eosin–stained skin biopsy of rash revealing nonnecrotic granuloma interspersed with degraded collagen fibers and lymphocytic infiltration. (Hematoxylin-eosin stain; original magnifications: **A,** 10×; **B,** 10×; **C**, 20×; **D,** 40×; **E,** 40×).

He had a remarkable medical history for myelodysplastic syndrome, atrial fibrillation, and chronic renal failure, with a baseline serum creatinine level of 2.0 mg/dL. Because of the indolent course of myelodysplastic syndrome, systemic therapy had not been initiated nor planned. The etiology of his chronic renal failure was not related to hypertension or diabetes. He had taken carvedilol, tamsulosin, and rivaroxaban for several years, and the eruption was not temporally related to any of the medications. Biopsy confirmed the suspected diagnosis of RGD, with the likely etiology being the patient’s underlying myelodysplastic syndrome (Fig 2).

The patient deferred systemic corticosteroids and was started on deucravactinib 6 mg daily. Clinical improvement was observed at 2 weeks. The medication was continued for 21 days until an elevation of serum creatinine level to 2.8 mg/dL (from a baseline of 2.0 mg/dL) was noted, and the medication was discontinued. After 1 month, the patient was clear of eruption at the follow up visit, without evidence of rebound (Fig 1, *B*).

## Discussion

RGD is a relatively recent umbrella term for multiple subtypes of granulomatous dermatitis, including interstitial granulomatous dermatitis, palisaded neutrophilic and granulomatous dermatitis, and interstitial granulomatous drug reactions. Given clinical, histologic, and etiologic similarities between these conditions, clinicians may find the use of the umbrella term useful.^4^ RGD is rarely a cutaneous manifestation of myelodysplastic syndrome and myeloid leukemia.^1^ For resolution of RGD due to this etiology, one option is treating the underlying myeloid disorder, in which case the treatment options must be weighed based on risk stratification to balance benefits vs adverse effects, especially if the disease is indolent.^5^ Other treatment options primarily involve topical steroids, which our patient did not respond to, and oral steroids, which were deferred.^2^ The coexisting renal disease also precluded methotrexate and cyclosporine.

With these limitations in treatment options, we considered TYK2 inhibition as an alternate therapeutic option. Upregulation of the JAK-STAT pathway, leading to aberrant inflammation, has been implicated in the formation of noninfectious granulomas, such as in sarcoidosis.^3^ Successful resolution of granulomatous skin conditions with JAK inhibitors has been reported.^6^ TYK2 is one member of the JAK family of proteins, making TYK2 a potential target for therapies countering inappropriate upregulation of JAK-STAT signaling pathways. Mouse models of human inflammatory conditions have demonstrated lower aberrant inflammation in *Tyk2* knockout mice.^7^ Of interest, *Tyk2* knockout mice also have decreased granuloma formation when exposed to granuloma-inducing stimuli, indicating that *Tyk2* may play a role in granuloma formation.^8^

Because of the previously reported success of JAK inhibition in treating granulomatous skin conditions combined with evidence that TYK2 inhibition could target granuloma formation, we decided to treat the patient with deucravacitinib. Deucravacitinib, an allosteric TYK2 inhibitor, was recently US Food and Drug Administration-approved for psoriasis, another condition with upregulated JAK-STAT signaling.^9^ Our report demonstrates the efficacy of systemic TYK2 inhibition with deucravacitinib in obtaining rapid clinical relief of the debilitating RGD associated with our patient’s myelodysplastic syndrome.

For our patient, we needed to terminate deucravacitinib therapy because of a significant increase in serum creatinine, indicating nephrotoxicity. It is unclear whether TYK2 inhibition in a patient without a previous history of chronic kidney disease would cause nephrotoxicity. Aside from nephrotoxicity considerations reported here, other common deucravacitinib adverse effects include nasopharyngitis, upper respiratory infections, and headache.^9^ Because specific targeting of TYK2 therapy leads to a narrow adverse effect profile and our patient demonstrated no other adverse events, we do not expect adverse interactions for TYK2 therapy specific to myelodysplastic syndrome. However, further research into safety of TYK2 therapy given comorbidities would be helpful.

Our findings also suggest that TYK2 may be a novel target for other noninfectious granulomatous disorders, such as granuloma annulare and sarcoidosis. Current therapies for these disorders have considerable adverse effect profiles because regimens using glucocorticoids with antimetabolites or anti–tumor necrosis factor-α therapy lead to increased risk of infection from immunosuppression.^10^ As a more selective inhibitor, TYK2 inhibition therapy could have fewer adverse events.^9^ Of note, effective treatment of cutaneous sarcoidosis with tofacitinib, a JAK inhibitor, has been noted in a case report, indicating a possible role for TYK2 inhibitors in this condition because common targets of JAK and TYK2, such as STAT3, are likely required for maintenance of granulomas.^3^

In summary, because of the efficacy of TYK2 inhibition in resolving RGD, more research is warranted regarding the potential of this therapy to treat noninfectious granulomatous disorders, including potential cutaneous manifestations of myelodysplastic syndrome. Improved understanding of the nephrotoxicity of TYK2 inhibitors can also help tailor decision making for therapies.

## Conflicts of interest

None.
